# Effects of Gender and Apolipoprotein E on Novelty MMN and P3a in Healthy Elderly and Amnestic Mild Cognitive Impairment

**DOI:** 10.3389/fnagi.2018.00256

**Published:** 2018-08-21

**Authors:** Lijuan Gao, Jiu Chen, Lihua Gu, Hao Shu, Zan Wang, Duan Liu, Yanna Yan, Zhijun Zhang

**Affiliations:** ^1^Department of Neurology, Affiliated Zhongda Hospital, School of Medicine, Southeast University, Nanjing, China; ^2^Department of Psychology, Xinxiang Medical University, Xinxiang, China

**Keywords:** apolipoprotein E, gender, event-related potential, amnestic mild cognitive impairment, novelty mismatch negativity, novelty P3a

## Abstract

**Background:** The apolipoprotein E epsilon4 (*ApoE* ε4) allele and female gender may be important risk factors for the development of Alzheimer’s disease and amnestic mild cognitive impairment (aMCI). Novelty mismatch negativity (MMN) represents the pre-attentive index of deviance detection and P3a represents the attention orienting response. Furthermore, MMN and P3a components have been reported to be potential markers in aMCI. Therefore, this study will investigate the effects of gender and *ApoE* on auditory novelty MMN and P3a and their relationship to neuropsychological performance in aMCI.

**Methods:** Thirty nine aMCI subjects and 44 controls underwent neuropsychological assessment and *ApoE* genotyping. Novelty MMN and P3a components were investigated during an auditory novelty oddball task.

**Results:** Firstly, novelty MMN latency was significantly shorter in aMCI than in healthy control (HC) group. Secondly, novelty MMN latency was negatively correlated with episodic memory in aMCI, but not in HC. Novelty P3a latency was negatively correlated with information processing speed in all subjects. For gender effect, novelty MMN latency was shorter in aMCI females than in HC females. Moreover, novelty P3a amplitudes were lower in males than in females in both aMCI and HC. For the effect of *ApoE* status, novelty MMN latency was shorter in aMCI *ApoE* ε4- than HC *ApoE* ε4-.

**Conclusion:** aMCI presents altered pre-attentive processing indexed by novelty MMN components. Furthermore, there may be a compensatory mechanism on the impaired processing in aMCI. It further suggests that aMCI female and *ApoE* ε4- recruited the compensatory mechanism.

## Introduction

Amnestic mild cognitive impairment (aMCI) is thought to reflect a transitional state between normal aging and dementia due to Alzheimer’s disease (AD) (Petersen and Negash, 2008; Albert et al., 2011). Behaviorally, both AD and aMCI are traditionally characterized and diagnosed in association with disruption in higher-level brain functions such as memory, perception, executive function, and attention. More specifically, abnormalities of episodic memory and attentional functions are the earliest clinical symptoms of aMCI and AD (Sperling et al., 2011; Simon et al., 2012; Bourrelier et al., 2015). Therefore, it is important to determine whether the abnormal mechanism of attentional function can contribute to the search for markers of the disease process early in the course of AD.

Event-related potential (ERP) measurements, a powerful non-invasive approach with a time resolution of milliseconds, is widely utilized to assess information processing of different cognitive functions in individuals (Kim et al., 2013). Mismatch negativity (MMN) and P300 subcomponent (P3a) are thought to be indices of the pre-attentive information processing (Naatanen et al., 2012) and attention orienting process (Jaworska et al., 2013), respectively. It was reported that both MMN and P3a components can be simultaneously elicited by any discriminable changes (including novel sounds) in a passive oddball experimental paradigm (Fisher et al., 2014). The novelty P3a component is considered to follow MMN and derives from fronto-central lobes during task processing and the negative novelty MMN component is thought to be located in the frontal and temporal regions and be associated with a mismatch between a trace in perceptual and sensory memory inputs (Naatanen et al., 2007). Recently, several studies suggested the combination of MMN and P3a (MMN/P3a complex) might be an underlying index of fundamental perceptual and pre-attentive information processing in the auditory pathway (Hermens et al., 2010; Chen et al., 2015). Furthermore, a recent study has indicated that novelty MMN is a more sensitive biomarker for aMCI than conventional MMN (Lindin et al., 2013). Therefore, the novelty MMN/P3a complex may be a useful brain marker to estimate cortical markers in the underlying neurobiology of aMCI.

Growing evidence from diverse aspects suggests that female gender may be an important risk factor for the development of AD and aMCI. Evidence from epidemiological studies has indicated a higher prevalence of AD in females than in age-matched males (Mielke et al., 2014). Previous studies have reported a faster rate of general cognitive function decline in females compared to males, with an average additional decline of 3.8 points over 5 years (Tschanz et al., 2011). Furthermore, a cross-sectional research study has reported females with AD had smaller hippocampal volumes than males (Apostolova et al., 2006) and a longitudinal study showed that the temporal lobe atrophic rate was about 1–1.5% faster in AD women than in men (Hua et al., 2010). Several studies investigated gender effects on components in auditory oddball paradigms in healthy subjects. For MMN, compared to males, females exhibited larger MMN amplitude in healthy young subjects (Barrett and Fulfs, 1998). In contrast, some studies reported no gender effects on the amplitude or latency of MMN in healthy young and old subjects (Kasai et al., 2002; Tsolaki et al., 2015; Yang et al., 2016). For P3, compared to males, females exhibited larger P3b amplitude and shorter latency in healthy young subjects (Yuan et al., 2008). In contrast, one study reported no gender effects on the amplitude or latency of P3b in healthy young and old subjects (Tsolaki et al., 2015). However, gender effect on novelty MMN and P3a in aMCI was not reported.

Apolipoprotein E (ApoE) is a glycoprotein of 299 amino acids with a molecular mass of ∼34 kDa and is encoded by a polymorphic gene localized on chromosome 19. The *ApoE* gene exists as three alleles (ε2, ε3, and ε4), which encode for ApoE2, ApoE3, and ApoE4, respectively (Kanekiyo et al., 2014). The apolipoprotein E epsilon4 (*ApoE* ε4) allele is the major genetic susceptibility factor for the development of AD (Teter et al., 2002). Individuals with the ε4 allele have an increased risk of developing AD and decreased age of onset (Raber et al., 2004). *ApoE* ε4 carriers have a greater rate of cognitive decline compared with non-carriers (Olofsson et al., 2010). There were two studies regarding the effects of *ApoE* status on components in auditory oddball paradigms in mild cognitive impairment patients. One study showed no significant differences in MMN, P3a and P3b indices between *ApoE* ε4 carriers and non-carriers in mildly cognitively impairment patients (Reinvang et al., 2005). The subjects of this research combined MCI and subjective cognitive complaints without neuropsychological deficits. Another study revealed that *ApoE* ε4 carriers with MCI had longer P3b latency than non-carriers (Barcin and Cintra, 2018). The effect of *ApoE* on novelty MMN and P3a in pure aMCI has not been reported.

The objective of the present study was thus to assess the diagnostic value of novelty MMN and P3a, and investigate the effect of gender and *ApoE* ε4 on auditory novelty MMN and P3a in healthy control (HC) and aMCI.

## Materials and Methods

### Subjects

The present study recruited 83 elderly individuals (all of whom were Chinese Han; right-handed; age range: 55–80 years old; ≥8-year education), including 39 aMCI patients and 44 HC, through newspaper advertisements, normal community health screening, and a hospital outpatient service. Written informed consent was obtained from all participants, and the study was approved by the responsible Human Participants Ethics Committee of Affiliated Zhongda Hospital, Southeast University (2016ZDSYLL011.0). All aMCI-multiple domain subjects met the diagnostic criteria proposed by Petersen (2004) and others’ recommendations (Albert et al., 2011) including: (1) subjective memory impairment corroborated by subject and an informant; (2) objective memory performances documented by an Auditory Verbal learning Test-delayed recall (AVLT-DR) score that is ≤1.5 standard deviation (SD) of age- and education-adjusted norms; (3) normal general cognitive function evaluated by a Mini-Mental State Examination (MMSE) score of 24 or higher; (4) a Clinical Dementia Rating of 0.5, with at least a 0.5 score in the memory domain; (5) no or minimal impairment in daily living activities; and (6) absence of dementia, or does not meet the Diagnostic and Statistical Manual of Mental Disorders-IV (DSM-IV) criteria and the National Institute on Aging-Alzheimer’s Association (NIA-AA) workgroups on diagnostic guidelines for AD. Exclusion criteria were as follows: (1) history of stroke, alcohol, or drug abuse/dependence, traumatic brain injury, Parkinson’s disease, epilepsy; (2) major medical or psychiatric illnesses (e.g., cancer, anemia, thyroid dysfunction, and major depression); and (3) severe visual or hearing loss.

Healthy control were required to have a clinical dementia rating of zero, an MMSE score of ≥26, and an AVLT-DR score >4. These participants were matched for age, gender, and level of education by subject to aMCI. The inclusion and exclusion assessment was performed by two experienced neuropsychiatric physicians who administered a structured interview to subjects and their informants (Chen et al., 2016c).

### Neuropsychological Assessment

All subjects underwent a standardized clinical interview and comprehensive neuropsychological assessments performed by neuropsychologists (Drs. Gu, Gao, and Yan). The neuropsychological assessments included cognitive function as follows (Chen et al., 2016a,c): (1) general cognitive function: MMSE and Mattis Dementia Rating Scale 2 (MDRS-2); (2) episodic memory: Auditory Verbal Learning Test (AVLT), Rey-Osterrieth Complex Figure Test-Delayed Recall (ROCFT-DR), and Logical Memory Test-Delayed Recall (LMT-DR); (3) information processing speed: Trail-Making Test A (TMT-A), Digital Symbol Substitution Test (DSST), Stroop Color and Word Tests A and B (Stroop-A and Stroop-B); (4) executive function: Verbal Fluency Test (VFT), Digit Span Test (DST), Trail-Making Test B (TMT-B), Stroop Color and Word Test C (Stroop-C), and Semantic Similarity Test (Similarity); and (5) visuospatial function: Clock Drawing Test (CDT) and Rey-Osterrieth Complex Figure Test (ROCFT).

### *ApoE* Genotyping

A DNA purification kit (Tiangen, China) was used to extract genomic DNA from EDTA-anticoagulated blood. *ApoE* genotypes were determined by polymerase chain reaction-based restriction fragment length polymorphism (PCR-RFLP) (Chen et al., 2016b).

### Procedure and Stimuli

After preparation for electroencephalogram (EEG) recording, participants were presented, via headphones, with 280 binaural pure tones (80 dB SPL, 10 ms rise/fall) at a 200 ms stimulus onset asynchrony; this comprised a pseudo-random sequence of 224 (80%) 1000 Hz standard tones, 28 (10%) 1050 Hz deviant tones, and 28 (10%) novel stimuli. Tones were presented while participants watched a silent video of a comedy movie. Participants were asked to report back the storyline of the movie at the end of the task.

### EEG Acquisition and Analysis

EEG measurements of 64 scalp locations based on the 10–20 system were recorded using a BrainAmp MR portable ERP system (Brain Products GmbH; Munich, Germany). Data was referenced to a common average reference. Blinks and eye movement artifacts were monitored by recording the vertical and horizontal electro-oculogram located above and below the midpoint of the right eye and the outer canthus of each eye. Ocular artifact correction was performed using independent component analysis (Jung et al., 2000; Neuhaus et al., 2011). The EEG was band-pass filtered from 0.1 to 100 Hz with a gain of 20,000. EEG signals were analyzed with Brain Vision Analyzer software (Brain Products GmbH). In offline analyses, the EEG signal was band-pass filtered at 0.01–30 Hz (Chen et al., 2014a,b, 2015). EEG signals with an amplitude larger than ±100 μV were rejected (Chen et al., 2015). Mismatched difference waveforms were acquired by subtracting ERP waveforms induced by the novel auditory stimulus from those of the standard auditory stimulus. To calculate the ERP, epochs of EEG were averaged offline from 100 ms pre-stimulus to 700 ms post-stimulus relative to a 100 ms pre-stimulus baseline. The times were relative to the stimulus onset. The peak amplitudes and latencies of novelty MMN and P3 were identified within the established epochs of 150–250 and 250–450 ms, respectively (Jia et al., 2015; Weigl et al., 2016) and acquired with peak detection process. Novelty MMN and P3 variables were acquired at fronto-central (Fz, FCz, Cz) electrodes.

### Statistical Analysis

Statistical analyses were conducted with SPSS 18.0 software. The Student *t*-test and chi-square test were performed to compare the demographic data and neuropsychological performances between aMCI group and HC group.

Mean peak amplitudes and latencies were subjected to repeated measures ANOVA (RMANOVA) with Group (aMCI and HC) as one between-subjects factor, and Electrode location (Fz, FCz, Cz) as one within-subject factor for novelty MMN and P3a. Years of education were included as covariates. To investigate the effect of gender on novelty MMN and P3, mean peak amplitudes and latencies were subjected to ANOVA among four groups (aMCI female, aMCI male, HC female, HC male) in Fz, FCz, Cz electrode sites. To investigate the effect of *ApoE* status on novelty MMN and P3, mean peak amplitudes and latencies were subjected to ANOVA among four groups (aMCI *ApoE* ε4+, aMCI *ApoE* ε4-, HC *ApoE* ε4+, HC *ApoE* ε4-) in Fz, FCz, Cz electrode sites. Degrees of freedom were corrected for non-sphericity using the Greenhouse–Geisser adjustment. *Post hoc* comparisons were analyzed by LSD test.

Partial correlation analyses were applied between amplitudes and latencies of novelty MMN and P3a and the neuropsychological performances in aMCI, HC and all subjects, controlling for the effects of age, gender, education years, *ApoE* status, and group. To increase statistical power by reducing random variability, as previously described (Shu et al., 2016), we grouped the neuropsychological tests into four cognitive domains and transformed the raw scores into four composite Z scores.

## Results

### Demographic and Neuropsychological Characteristics

The demographic and neuropsychological characteristics for all subjects are shown in **Table 1** and **Supplementary Table S1**. No significant differences in age, gender or *ApoE* status were found between aMCI and HC (*p* > 0.05). Compared with HC, aMCI showed significant declines in years of education, MMSE, MDRS-2, information processing speed function, executive function, episodic memory and visuospatial function (*p* < 0.05).

**Table 1:** Demographics and clinical measures of subjects with aMCI and HC subjects.

Items	HC (*N* = 44)	aMCI (*N* = 39)	*t*-Values (χ^2^)	*p*-Values
Age (years)	69.93 (5.58)	71.28 (5.98)	-1.06	0.290
Gender (males/females)	21/23	25/14	2.24	0.134
Education (years)	12.23 (2.95)	10.81 (2.93)	2.19	0.031^∗^
*ApoE* ε4 carriers (%)	18 (40.91%)	12 (30.77%)	0.92	0.337
MMSE score	28.41 (1.31)	27.08 (2.11)	3.49	0.001^∗^
MDRS-2 score	137.59 (3.17)	134.85 (5.35)	2.80	0.007^∗^
Episodic memory Z score	0.49 (0.53)	-0.54 (0.61)	8.19	0.000^∗^
Information processing speed Z score	0.21 (0.82)	-0.21 (0.64)	2.61	0.011^∗^
Executive function Z score	0.25 (0.64)	-0.24 (0.50)	3.83	0.000^∗^
Visuospatial function Z score	0.26 (0.45)	-0.31 (0.82)	3.81	0.000^∗^


*aMCI, amnestic mild cognitive impairment; HC, healthy controls; MMSE, Mini Mental State Exam; MDRS-2, Mattis Dementia Rating Scale 2. ^∗^Significant differences were found between aMCI patients and HC subjects. *p*-Values were obtained by Student’s *t*-test except for gender and ApoE ε4 carriers (chi square test).*

### Novelty MMN

For novelty MMN latency, RMANOVA showed significant effects of the Group factor [*F*_(2,79)_ = 4.796, *p* = 0.031], but no significant Electrode Sites factor [*F*_(1,80)_ = 0.331, *p* = 0.681]. Compared with HC (mean = 204.33 ± 27.82 ms), aMCI (mean = 190.72 ± 26.45 ms) had shorter novelty MMN latency. For novelty MMN amplitude, RMANOVA showed significant effects of the Electrode Sites factor [*F*_(2,79)_ = 3.673, *p* = 0.042], but there was no significant Group factor [*F*_(1,80)_ = 0.000, *p* = 0.996].

#### Effect of Gender

There was a significant effect of gender with shorter novelty MMN latency observed for aMCI female compared with HC female in Cz site (**Table 2**). No significant gender effect was found for novelty MMN amplitude (**Table 2**).

**Table 2:** Event-related potential (ERP) data for HC and aMCI with different gender.

Items	Site	HC		aMCI		*F*-Values	*p*-Values
					
		Male (*N* = 21)	Female (*N* = 23)	Male (*N* = 25)	Female (*N* = 14)		
Novelty MMN latency	Fz	197.24 (28.35)	203.39 (32.98)	197.44 (34.16)	199.71 (32.06)	0.18	0.908
	FCz	200.86 (35.51)	216.87 (25.49)	201.20 (34.96)	187.43 (42.51)	2.22	0.092
	Cz	201.43 (39.88)	205.04 (33.43)	180.56 (44.80)	172.43 (43.44)d	2.93	0.039*
Novelty MMN amplitude	Fz	-3.10 (1.78)	-4.01 (1.97)	-3.33 (1.76)	-3.30 (2.03)	1.04	0.379
	FCz	-2.65 (1.67)	-3.86 (1.56)	-3.02 (2.13)	-2.50 (1.87)	2.17	0.098
	Cz	-1.93 (1.17)	-2.77 (1.21)	-2.73 (2.52)	-2.21 (2.00)	1.62	0.191
Novelty P3 latency	Fz	364.47 (35.66)	358.87 (32.88)	370.88 (34.65)	377.71 (29.64)	1.47	0.230
	FCz	385.14 (39.81)	358.78 (28.15)	358.16 (47.23)	370.29 (33.88)	2.03	0.116
	Cz	390.57 (43.77)	387.57 (39.13)	381.60 (51.33)	397.29 (44.48)	0.40	0.755
Novelty P3 amplitude	Fz	3.52 (1.65)	5.56 (2.86)a	3.57 (2.45)	5.60 (2.88)b	4.44	0.006*
	FCz	3.22 (1.47)	5.07 (2.93)a	3.23 (3.05)	5.16 (2.59)b	3.79	0.014*
	Cz	2.49 (1.08)	3.78 (2.31)	2.74 (2.89)	3.83 (2.32)	2.36	0.078


*aMCI, amnestic mild cognitive impairment; HCs, healthy controls. ^∗^Significant differences were found among the four groups. *p*-Values were obtained by ANOVA analysis. ^*a,b,d*^*Post hoc* analysis (LSD test) further revealed the source of ANOVA difference (a, HC male vs. HC female; b, aMCI male vs. aMCI female; d, aMCI female vs. HC female).*

#### Effect of *ApoE* Status

There was a significant effect of *ApoE* status with shorter novelty MMN latency observed for aMCI *ApoE* ε4- compared with HC *ApoE* ε4- (**Table 3**). No significant *ApoE* status effect was found for novelty MMN amplitude (**Table 3**).

**Table 3:** Event-related potential (ERP) data for HC and aMCI subjects with different *ApoE* status.

Items	Site	HC		aMCI		*F*-Values	*p*-Values
					
		*ApoE* ε4+ (*N* = 18)	*ApoE* ε4- (*N* = 26)	*ApoE* ε4+ (*N* = 12)	*ApoE* ε4- (*N* = 27)		
Novelty MMN latency	Fz	206.67 (29.85)	196.15 (31.04)	190.00 (42.07)	201.93 (28.25)	0.81	0.490
	FCz	215.78 (28.25)	204.69 (33.13)	198.50 (30.26)	195.26 (41.31)	1.34	0.266
	Cz	203.67 (35.83)	203.08 (37.26)	187.00 (31.75)	173.48 (48.31)d	3.11	0.031*
Novelty MMN amplitude	Fz	-4.01 (2.20)	-3.27 (1.67)	-3.50 (1.71)	-3.24 (1.91)	0.74	0.534
	FCz	-3.83 (2.17)	-2.90 (1.21)	-2.93 (1.84)	-2.80 (2.14)	1.28	0.288
	Cz	-2.50 (1.29)	-2.28 (1.24)	-2.65 (1.69)	-2.49 (2.60)	0.12	0.945
Novelty P3 latency	Fz	351.89 (27.84)	368.23 (36.64)	378.17 (30.40)	371.19 (34.01)	1.85	0.144
	FCz	365.78 (35.05)	375.23 (37.37)	368.83 (42.24)	359.70 (43.62)	0.69	0.562
	Cz	379.67 (47.44)	395.46 (35.31)	384.50 (50.53)	388.44 (49.21)	0.47	0.706
Novelty P3 amplitude	Fz	3.99 (2.00)	4.99 (2.84)	4.28 (3.24)	4.31 (2.58)	0.58	0.629
	FCz	3.11 (1.92)	4.93 (2.62)	3.02 (2.39)	4.33 (3.21)	2.38	0.076
	Cz	2.40 (1.71)	3.70 (1.91)	2.71 (2.09)	3.32 (2.98)	1.32	0.275


*aMCI, amnestic mild cognitive impairment; HCs, healthy controls. ^∗^Significant differences were found among the four groups. *p*-Values were obtained by ANOVA analysis. ^*d*^*Post hoc* analysis (LSD test) further revealed the source of ANOVA difference (d, aMCI ApoE ε4- vs. HC ApoE ε4-).*

### Novelty P3a

For novelty P3a amplitude, RMANOVA showed significant effects of the Electrode Sites factor [*F*_(2,79)_ = 19.840, *p* < 0.000], but no significant Group factor [*F*_(1,80)_ = 0.029, *p* = 0.864]. For novelty P3a latency, RMANOVA showed no significant effects of the Electrode Sites factor [*F*_(2,79)_ = 1.469, *p* = 0.234] and Group factor [*F*_(1,80)_ = 0.250, *p* = 0.619].

#### Effect of Gender

There was a significant effect of gender with lower novelty P3a amplitudes observed for males compared with females in both aMCI and HC in FCz and Fz sites (**Table 2**). No significant gender effect was found for novelty P3a latency (**Table 2**).

#### Effect of *ApoE* Status

No significant *ApoE* status effect was found for novelty P3a amplitudes and latencies (**Table 3**).

### The Relationship Between Key Clinical Variables, Neuropsychological Performance, and ERP Indices

**FIGURE 1:**
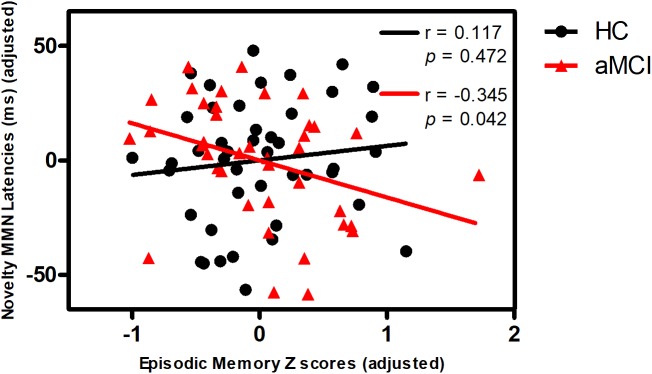
Correlation of novelty MMN latency with episodic memory in aMCI and HC. Scattergram represents correlation between episodic memory Z score (adjusted scores) and novelty MMN latency (adjusted scores) in HC (black line) and aMCI (red line). Note that the data of the latency is mean latency from the Fz, FCz, Cz electrodes. The figure used the adjusted data controlling for the effects of age, gender, education years, *ApoE* status, and group.

**FIGURE 2:**
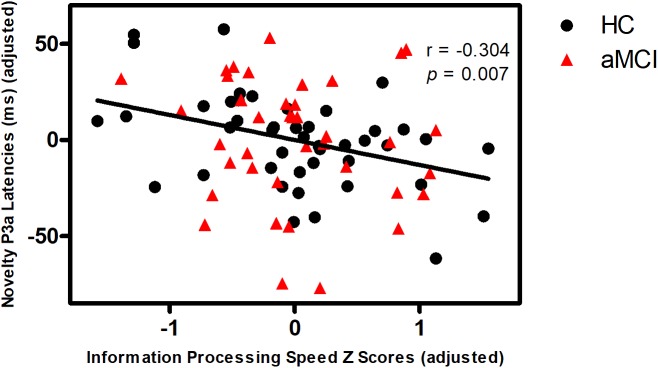
Correlation of novelty P3a latency with information processing speed in all subjects. Scattergram represents correlation between information processing speed Z score (adjusted scores) and novelty P3a latency (adjusted scores) in all subjects (black line). Note that the data of the latency is mean latency from the Fz, FCz, Cz electrodes. The figure used the adjusted data controlling for the effects of age, gender, education years, *ApoE* status, and group.

Partial correlation analyses demonstrated that novelty MMN latency was negatively correlated with episodic memory in aMCI, but not in HC (**Figure 1**). Novelty P3a latency was negatively correlated with information processing speed in all subjects (**Figure 2**). No correlations were found between MMN and P3a amplitudes and latencies and other neuropsychological performances in aMCI and HC (*p* > 0.05).

## Discussion

The current study is the first to examine the effect of gender and *ApoE* status on novelty MMN and P3a in pure aMCI. In summary, novelty MMN latency was significantly shorter in aMCI than in the HC group. Novelty MMN latency was negatively correlated with episodic memory in aMCI, but not in HC. Novelty P3a latency was negatively correlated with information processing speed in all subjects. For gender effect, novelty MMN latency was shorter in aMCI female than in HC female. Moreover, novelty P3a amplitudes were lower in male than in female in both aMCI and HC. For the effect of *ApoE* status, novelty MMN latency was shorter in aMCI *ApoE* ε4- than HC *ApoE* ε4-.

### Novelty MMN

Novelty MMN latency was significantly shorter in aMCI than in the HC group, which is consistent with one previous study (Lindin et al., 2013). This may represent a compensatory mechanism in aMCI. Studies have reported increased activity in the prefrontal lobe in MCI group (Browndyke et al., 2013), and increased functional connectivity between the right prefrontal regions and other regions in aMCI group (Bai et al., 2009). In addition, MCI has shown significantly higher EEG synchronization in the alpha and beta frequency ranges, but are no longer effective once AD develops, which indicated compensational mechanisms in MCI (Pijnenburg et al., 2004). Therefore, shorter novelty MMN latency may suggest a compensation mechanism of frontal cortex in aMCI. However, Ji et al. (2015) observed longer MMN latency in aMCI than in HC group, which may be attributed to relatively low years of education (aMCI mean = 3.88 ± 2.80, HC mean = 4.90 ± 2.76) of patients in the study, while years of education were higher in this research (aMCI mean = 10.81 ± 2.93, HC mean = 12.23 ± 2.95) and the research of Lindin et al. (2013) (aMCI mean = 10.15 ± 4.7, HC mean = 9.4 ± 4.4), respectively. One study has observed that glucose metabolism was positively associated with years of education in frontal lobe in MCI patients, suggesting a compensatory increase (Arenaza-Urquijo et al., 2017). Therefore, novelty MMN latency showed inconsistent changes in aMCI patients in these studies.

In addition, Novelty MMN latency was negatively associated with episodic memory in aMCI, indicating that novelty MMN latency may predict episodic memory performance in aMCI. Functional magnetic resonance imaging studies have reported association between episodic memory and functional connectivity between the prefrontal regions and other regions in aMCI group (Xie et al., 2012; Yuan et al., 2016). Although shorter novelty MMN latency represents a compensatory mechanism, shorter novelty MMN latency still reflects better episodic memory in aMCI.

Interestingly, this study showed that novelty MMN latency was shorter in aMCI female than in HC female, but not male, indicating that there is a gender effect on the altered MMN indices, that is, only females with aMCI present the altered pre-attentive processing. Accordingly, the findings further corroborated previous studies (Tschanz et al., 2011; Mielke et al., 2014), which suggest that females with aMCI may be more susceptible to AD pathology than males. One reason is that the effects of the ε4 genotype are more pronounced in women than in men. Some studies have reported that women with one ε4 allele have about a fourfold risk of AD (Farrer et al., 1997). Second reason is that the *ApoE* ε4 allele also has a greater deleterious effect in women than in men. Some studies have reported that, compared with men at different stages of AD, female with *ApoE* ε4 allele showed a greater deleterious effect on memory performance, cortical thickness, hippocampal pathology, functional connectivity changes in the default mode network (Fleisher et al., 2005). Furthermore, a large autopsy study found that women with ε4 carriers had greatest amyloid plaque and neurofibrillary tangle pathology (Corder et al., 2004). Collectively, based on the above-mentioned findings in aMCI, it further suggests that aMCI female recruit a compensatory mechanism, and female gender should clinically be considered as an important risk factor for the development of AD and aMCI, especially in clinical trials.

Additionally, novelty MMN latency was shorter in aMCI *ApoE* ε4- than HC *ApoE* ε4-, but not *ApoE* ε4+, suggesting that aMCI *ApoE* ε4- recruit a compensatory mechanism. One previous study also indicated that aMCI *ApoE* ε4- are able to compensate for reduced processing efficiency by recruiting distributed cortical areas through alpha and beta oscillatory cortical networks while aMCI *ApoE* ε4+ do not recruit compensatory mechanisms (Prieto del Val et al., 2015). No significant effect of *ApoE* status was found on MMN amplitude or latency in aMCI. The findings were consistent with a previous study (Reinvang et al., 2005), which showed no significant difference of novelty MMN potentials among *ApoE* gene status in mildly cognitive impaired patients, although subjects of the study combined MCI and subjective cognitive complaints without neuropsychological deficits. Furthermore, the current study demonstrated no significant difference of novelty MMN potentials between *ApoE* ε4+ and *ApoE* ε4- in HC. These results suggest that *ApoE* status does not affect novelty MMN potentials in HC or aMCI.

### Novelty P3a

This study showed no significant difference in novelty P3a amplitude or latency between aMCI and HC, which indicates that aMCI have no deficits on attention orienting response. However, novelty P3a latency was negatively correlated with information processing speed in all subjects. According to the theoretical models of attention, attention has many aspects, including arousal, selection, strategic control, and processing speed (Ponsford et al., 2014). Furthermore, several studies have reported that P3a represents the attention orienting response (Jaworska et al., 2013). Therefore, the findings suggest that novelty P3a latency can reflect the performance of information processing speed.

For gender effect, novelty P3a amplitudes were lower in male than in female in both aMCI and HC, indicating that female recruited more attention orienting process than male during the novelty condition and the gender effect was not influenced by disease state. Furthermore, no significant effect of *ApoE* status was found on novelty P3a amplitude or latency in HC and aMCI, which corroborated previous finding that no significant differences in P3a indices between *ApoE* ε4+ and *ApoE* ε4- in mildly cognitively impairment patients (Reinvang et al., 2005). The result indicates that *ApoE* status does not affect novelty P3a potentials in HC or aMCI.

### Limitations

Although this is the first and largest study of novelty MMN/P3a in aMCI, the sample size was still relatively small, which might influence the explanation of the results. Due to the small sample size and only three *ApoE* ε2 carriers in aMCI, this study did not explore *ApoE* ε2 and ε3 carriers, respectively, and the *ApoE* alleles-by-gender interaction in aMCI. Future study will enlarge the sample size and further explore the effect of the three alleles (ε2, ε3, ε4) on novelty MMN/P3a in aMCI and the *ApoE* alleles-by-gender interaction in aMCI. In addition, the cross-sectional study design itself has limitations. The follow-up of this sample and an additional AD group will help to clarify whether these changes of pre-attentive information processing in aMCI represent a transitional state between normal aging and AD.

## Conclusion

The aMCI patients present altered pre-attentive processing indexed by novelty MMN components. Furthermore, there may be a compensatory mechanism on the impaired processing in aMCI. It further suggests that aMCI female and *ApoE* ε4- recruited the compensatory mechanism. Collectively, the present study may aid to focus attention to MMN and P3a indices when developing new potential biomarkers for the early identification of AD. Moreover, these findings underline the importance of taking into account the effects of sex and *ApoE* genotype in future clinical trials. The present results could help in identifying different aMCI phenotypes, and then to select possible target groups for prevention, and to design rational strategies for therapeutic trials.

## Author Contributions

LjG, LhG, and JC were in charge of EEG recording and ERP data acquisition and analysis. HS, ZW, DL, and YY were in charge of patient enrollment and neuropsychological assessments. LjG and ZZ had the major responsibility for preparing the paper and manuscript writing. ZZ and JC contributed to the design and plan of the study. ZZ supervised the project.

## Conflict of Interest Statement

The authors declare that the research was conducted in the absence of any commercial or financial relationships that could be construed as a potential conflict of interest.
