# Supplementary material for: Effects of Gender and Apolipoprotein E on Novelty MMN and P3a in Healthy Elderly and Amnestic Mild Cognitive Impairment
Source: Front Aging Neurosci. 2018 Aug 21;10:256. doi: 10.3389/fnagi.2018.00256 (PMC6110901; doi:10.3389/fnagi.2018.00256)
Supplement: Supplementary file 1 [file Table_1.docx]

Supplementary Material

# Effects of gender [and apolipoprotein E on novelty MMN and P3a in healthy elderly and amnestic mild cognitive impairment](http://xueshu.baidu.com/s?wd=paperuri%3A%284eca036f24837565f615301cfad9c5e1%29&filter=sc_long_sign&sc_ks_para=q%3DAPOE%20E4%20affects%20spatial%20working%20memory%20and%20attention%20in%20patients%20with%20amnestic%20mild%20cognitive%20impairment&sc_us=6640347938210534022&tn=SE_baiduxueshu_c1gjeupa&ie=utf-8)

**Lijuan Gao^1^, Jiu Chen^1*^, Lihua Gu^1^, Hao Shu^1^, Zan Wang^1^, Duan Liu^1^, Yanna Yan^2^, Zhijun Zhang ^1, 2*^**

^1^ Department of Neurology, Affiliated ZhongDa Hospital, School of Medicine, Southeast University, Nanjing, Jiangsu, 210009, China

^2^ Department of Psychology, Xinxiang Medical University, Xinxiang, Henan, 453003, China

*** Correspondence:** Corresponding Author: Zhijun Zhang, E-mail: [janemengzhang@vip.163.com](mailto:janemengzhang@vip.163.com); Jiu Chen, E-mail: [ericcst@aliyun.com](mailto:ericcst@aliyun.com)

**Table S1 Neuropsychological data for aMCI and HC subjects**

| Items | HC  (N=44) | aMCI  (N=39) | t values | *p* values |
| --- | --- | --- | --- | --- |
| **MDRS-2 raw scores** | | | | |
| Attention | 36.36 (0.81) | 36.08 (0.93) | 1.50 | 0.14 |
| Initiation/Preservation | 36.09 (1.72) | 35.13 (3.27) | 1.65 | 0.11 |
| Construct | 5.91 (0.29) | 5.90 (0.31) | 0.18 | 0.86 |
| Conceptual | 37.64 (1.67) | 36.95 (2.37) | 1.54 | 0.13 |
| Memory | 21.59 (1.76) | 20.97 (2.02) | 1.49 | 0.14 |
| Total | 137.59 (3.17) | 134.85 (5.35) | 2.80 | 0.007* |
| **Episodic memory** | | | | |
| AVLT-20min DR (raw score) | 6.05 (1.88) | 1.92 (1.71) | 10.41 | 0.000* |
| AVLT-20min DR (*Z* score) | 0.71 (0.69) | -0.79 (0.62) | 10.41 | 0.000* |
| LMT-20min DR (raw score) | 6.78 (2.32) | 4.14 (3.10) | 4.43 | 0.000* |
| LMT-20min DR (*Z* score) | 0.40 (0.77) | -0.47 (1.03) | 4.43 | 0.000* |
| CFT-20min DR (raw score) | 18.08 (6.70) | 12.35 (6.48) | 3.95 | 0.000* |
| CFT-20min DR (*Z* score) | 0.41 (0.94) | -0.40 (0.91) | 3.95 | 0.000* |
| **Visuospatial function** | | | | |
| CDT (raw score) | 9.30 (0.73) | 8.41 (1.33) | 3.68 | 0.001* |
| CDT (*Z* score) | 0.37 (0.65) | -0.42 (1.19) | 3.68 | 0.001* |
| CFT (raw score) | 35.41 (1.09) | 34.95 (1.62) | 1.54 | 0.13 |
| CFT (*Z* score) | 0.15 (0.80) | -0.19 (1.20) | 1.54 | 0.13 |
| **Information processing speed** | | | | |
| DSST (raw score) | 36.16 (10.24) | 31.81 (8.70) | 2.07 | .041* |
| DSST (*Z* score) | 0.22 (1.06) | -0.23 (0.90) | 2.07 | .041* |
| TMT-A (second) | 67.36 (20.36) | 69.05 (12.50) | -0.46 | 0.65 |
| TMT-A (*Z* score) | 0.15 (1.08) | -0.12 (0.76) | 1.32 | 0.19 |
| Stroop A (second) | 27.91 (8.90) | 30.38 (6.10) | -1.46 | 0.15 |
| Stroop A (*Z* score) | 0.27 (1.08) | -0.25 (0.84) | 2.43 | 0.017* |
| Stroop B (second) | 43.86 (13.00) | 47.65 (10.09) | -1.44 | 0.15 |
| Stroop B (*Z* score) | 0.21 (1.04) | -0.24 (0.94) | 1.99 | 0.05 |
| **Executive function** |  |  |  |  |
| VFT (raw score) | 24.16 (5.87) | 22.33 (6.45) | 1.35 | 0.18 |
| VFT (*Z* score) | 0.15 (0.96) | -0.15 (1.05) | 1.35 | 0.18 |
| DST-backward (raw score) | 4.82 (1.78) | 4.08 (1.33) | 2.13 | 0.04* |
| DST-backward (*Z* score) | 0.25 (1.11) | -0.21 (0.82) | 2.13 | 0.04* |
| TMT-B (second) | 164.50 (52.42) | 204.23 (67.77) | -3.01 | 0.004* |
| TMT-B (*Z* score) | 0.30 (1.00) | -0.30 (0.95) | 2.80 | 0.006* |
| Stroop C (second) | 81.82 (33.08) | 95.59 (32.28) | -1.89 | 0.06 |
| Stroop C (*Z* score) | 0.29 (1.08) | -0.34 (0.83) | 2.87 | 0.005* |
| Similarity (raw score) | 19.50 (4.99) | 17.33 (3.41) | 2.28 | 0.03* |
| Similarity (*Z* score) | 0.26 (1.13) | -0.24 (0.78) | 2.28 | 0.03* |

Data are presented as mean ± stand deviation (SD). Abbreviations: aMCI: amnestic mild cognitive impairment; HC: healthy controls; MDRS-2, Mattis Dementia Rating Scale-2; AVLT-20min DR, Auditory Verbal Learning Test-20-minute delayed recall; LMT-20min DR, Logical Memory Test-20-minute delayed recall; CFT-20min DR, Rey-Osterrieth Complex Figure Test-20-minute delayed recall; CDT, Clock Drawing Test; CFT, Rey-Osterrieth Complex Figure Test; DSST, Digital Symbol Substitution Test; TMT-A, Trail Making Test-A; Stroop, Stroop Color and Word Test; VFT, Verbal Fluency Test; DST, Digit Span Test; TMT-B, Trail Making Test-B; Similarity, Semantic Similarity Test.

*Significant differences were found among aMCI patients and HC subjects. *p* values were obtained by student t-test.
